# Bis(2,2′-diamino-4,4′-bi-1,3-thia­zole-κ^2^ *N* ^3^,*N* ^3′^)bis­(nitrato-κ*O*)lead(II) dihydrate

**DOI:** 10.1107/S1600536809029638

**Published:** 2009-07-29

**Authors:** Bing-Xin Liu, Duan-Jun Xu

**Affiliations:** a Department of Chemistry, Shanghai University, 200444 People’s Republic of China; b Department of Chemistry, Zhejiang University, Hangzhou, 310027, People’s Republic of China

## Abstract

In the title compound, [Pb(NO_3_)_2_(C_6_H_6_N_4_S_2_)_2_]·2H_2_O, the Pb^II^ cation is *N*,*N*′-chelated by two 2,2′-diamino-4,4′-bi-1,3-thia­zole (DABT) ligands and further is *cis* coordinated by two nitrate anions in a distorted PbN_4_O_2_ octa­hedral geometry. One of the uncoordinated water mol­ecules is close to an inversion center and is disordered equally over two sites. Intra­molecular N—H⋯N and N—H⋯O inter­actions are present. An extensive hydrogen-bonding network of types N—H⋯O, O—H⋯O, O—H⋯N and O—H⋯S consolidates the crystal structure.

## Related literature

For the application of 2,2′-diamino-4,4′-bi-1,3-thia­zole complexes as soft magnetic materials, see: Sun *et al.* (1997 ▶). For general background to the structures of complexes of 2,2′-diamino-4,4′-bi-1,3-thia­zole, see: Liu *et al.* (2003 ▶). For Pb—N bond distances in 2,2′-diamino-4,4′-bi-1,3-thia­zole complexes, see: Abedini *et al.* (2005 ▶); Liu *et al.* (2006 ▶). H atoms bonded to the disordered O atoms were placed in calculated positions, see: Nardelli (1999 ▶)
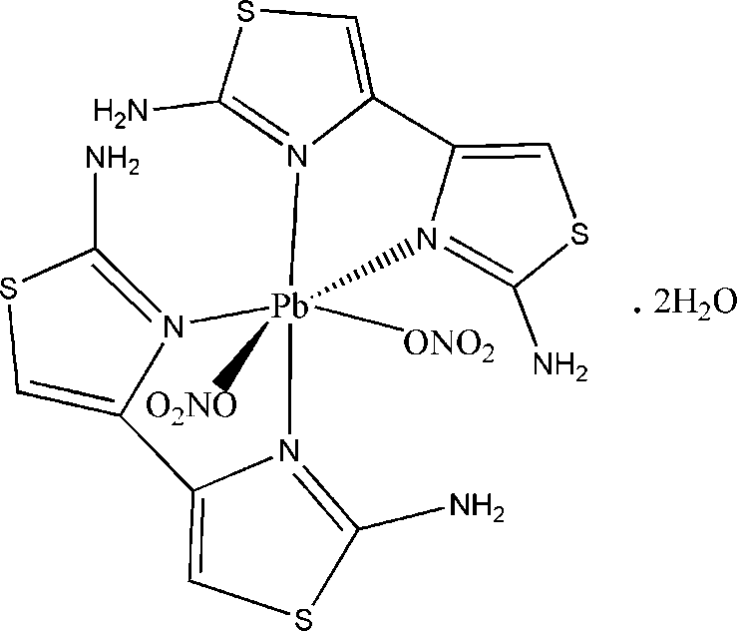


## Experimental

### 

#### Crystal data

[Pb(NO_3_)_2_(C_6_H_6_N_4_S_2_)_2_]·2H_2_O
*M* *_r_* = 1527.66
Triclinic, 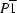

*a* = 9.2387 (8) Å
*b* = 9.6962 (9) Å
*c* = 13.5636 (6) Å
α = 105.731 (4)°
β = 90.377 (3)°
γ = 97.072 (5)°
*V* = 1159.61 (16) Å^3^
*Z* = 1
Mo *K*α radiation
μ = 7.70 mm^−1^
*T* = 294 K
0.21 × 0.16 × 0.14 mm

#### Data collection

Rigaku R-AXIS RAPID IP diffractometer
Absorption correction: multi-scan (*ABSCOR*; Higashi, 1995 ▶) *T*_min_ = 0.215, *T*_max_ = 0.340
6095 measured reflections
4012 independent reflections
3705 reflections with *I* > 2σ(*I*)
*R*_int_ = 0.015

#### Refinement

*R*[*F* ^2^ > 2σ(*F* ^2^)] = 0.025
*wR*(*F* ^2^) = 0.065
*S* = 1.08
4012 reflections
319 parameters
H-atom parameters constrained
Δρ_max_ = 0.83 e Å^−3^
Δρ_min_ = −0.46 e Å^−3^

### 

Data collection: *PROCESS-AUTO* (Rigaku, 1998 ▶); cell refinement: *PROCESS-AUTO*; data reduction: *CrystalStructure* (Rigaku/MSC, 2002 ▶); program(s) used to solve structure: *SIR92* (Altomare *et al.*, 1993 ▶); program(s) used to refine structure: *SHELXL97* (Sheldrick, 2008 ▶); molecular graphics: *ORTEP-3 for Windows* (Farrugia, 1997 ▶); software used to prepare material for publication: *WinGX* (Farrugia, 1999 ▶).

## Supplementary Material

Crystal structure: contains datablocks I, global. DOI: 10.1107/S1600536809029638/hk2747sup1.cif

Structure factors: contains datablocks I. DOI: 10.1107/S1600536809029638/hk2747Isup2.hkl

Additional supplementary materials:  crystallographic information; 3D view; checkCIF report

## Figures and Tables

**Table 1:** Selected bond lengths (Å)

Pb—N1	2.656 (4)
Pb—N3	2.563 (4)
Pb—N5	2.535 (5)
Pb—N7	2.692 (4)
Pb—O1	2.704 (4)
Pb—O4	2.803 (5)

**Table 2:** Hydrogen-bond geometry (Å, °)

*D*—H⋯*A*	*D*—H	H⋯*A*	*D*⋯*A*	*D*—H⋯*A*
N2—H2*A*⋯O1	0.92	2.08	2.884 (8)	145
N2—H2*B*⋯O4^i^	0.90	2.33	3.209 (7)	165
N2—H2*B*⋯O6^i^	0.90	2.31	3.057 (7)	140
N4—H4*A*⋯N7	0.99	2.19	3.168 (7)	166
N4—H4*B*⋯O1*W*^ii^	0.88	2.29	3.015 (8)	141
N4—H4*B*⋯O2*WA*^iii^	0.88	2.26	2.98 (9)	140
N6—H6*A*⋯N1	0.93	2.22	3.119 (8)	160
N6—H6*B*⋯O2*WA*^iv^	0.96	2.29	3.12 (10)	145
N6—H6*B*⋯O1*W*^iv^	0.96	2.10	2.929 (10)	144
N8—H8*A*⋯O3^v^	0.90	2.17	3.027 (7)	159
N8—H8*B*⋯O4	0.84	2.13	2.916 (7)	156
O1*W*—H1*A*⋯O3	0.85	1.94	2.782 (8)	168
O1*W*—H1*B*⋯O2*WA*	0.83	1.97	2.54 (9)	125
O1*W*—H1*B*⋯O2*WB*	0.83	2.14	2.93 (4)	160
O2*WA*—H2*C*⋯N4^iii^	0.85	2.42	2.98 (9)	124
O2*WA*—H2*D*⋯O1*W*^vi^	0.85	2.17	2.85 (9)	136
O2*WB*—H2*E*⋯S4^iii^	0.85	2.27	3.09 (5)	164
O2*WB*—H2*F*⋯S3^vii^	0.85	2.80	3.53 (5)	144
O2*WB*—H2*F*⋯N6^vii^	0.85	1.91	2.67 (5)	148

Symmetry codes: (i) 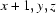; (ii) 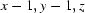; (iii) 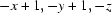; (iv) 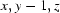; (v) 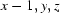; (vi) 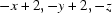; (vii) 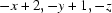.

**Fig. 1.**
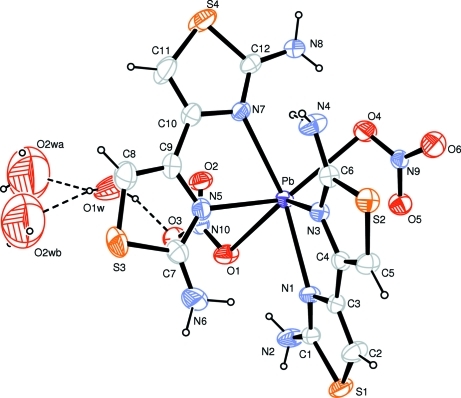
The molecular structure of the title compound with 30% probability displacement ellipsoids (arbitrary spheres for H atoms); dashed lines indicate the hydrogen bonding.

## supplementary crystallographic information

### Comment

Some metal complexes of 2,2'-diamino-4,4'-bi-1,3-thiazole (DABT) have shown potential application in the field of soft magnetic material (Sun *et al.*, 1997). As part of the ongoing structural investigation of metal complexes with DABT ligand (Liu *et al.*, 2003), the title Pb^II^ complex has recently been prepared and its crystal structure is reported herein.

In the title compound, the Pb^II^ cation is N,*N*'-chelated by two DABT ligands and further is *cis*-coordinated by two nitrate anions in a distorted PbN_4_O_2_ octahedral geometry (Fig. 1). The Pb—N bond distances (Table 1) are somewhat longer than those [2.527, 2.544 and 2.551 Å] found in other two Pb complexes with DABT ligand (Abedini *et al.* 2005; Liu *et al.* 2006). One of the lattice water molecules is close to an inversion center and is disordered equally over two sites. The extensive hydrogen bonding network of types N—H···O, O—H···O, O—H···N and O—H···S is present in the crystal structure.

### Experimental

An aqueous solution (15 ml) of DABT (0.20 g, 1 mmol) and Pb(NO_3_)_2_ (0.33 g, 1 mmol) was refluxed for 4 h. The solution was filtered after cooling to room temperature. Yellow single crystals were obtained from the filtrate after 4 d.

### Refinement

One of the lattice water molecules [O2W] is close to an inversion center and is disordered equally over two sites. H atoms bonded to the disordered O atoms are placed in calculated position (Nardelli, 1999). H atoms bonded to the O1W and N atoms were located in a difference Fourier map. All H atoms bonded to O and N atoms were refined as riding in as-found relative positions. Aromatic H atoms were placed in calculated positions with C—H = 0.93 Å and refined in riding mode. *U*_iso_(H) = 1.2*U*_eq_(carrier) for all H atoms.

### Crystal data

**Table:**

[Pb(NO_3_)_2_(C_6_H_6_N_4_S_2_)_2_]·2H_2_O	*Z* = 1
*M**_r_* = 1527.66	*F*(000) = 736
Triclinic, *P*1	*D*_x_ = 2.187 Mg m^−^^3^
Hall symbol: -P 1	Mo *K*α radiation, λ = 0.71073 Å
*a* = 9.2387 (8) Å	Cell parameters from 4246 reflections
*b* = 9.6962 (9) Å	θ = 2.2–25.0°
*c* = 13.5636 (6) Å	µ = 7.70 mm^−^^1^
α = 105.731 (4)°	*T* = 294 K
β = 90.377 (3)°	Block, yellow
γ = 97.072 (5)°	0.21 × 0.16 × 0.14 mm
*V* = 1159.61 (16) Å^3^	

### Data collection

**Table:**

Rigaku R-AXIS RAPID IP diffractometer	4012 independent reflections
Radiation source: fine-focus sealed tube	3705 reflections with *I* > 2σ(*I*)
graphite	*R*_int_ = 0.015
Detector resolution: 10.0 pixels mm^-1^	θ_max_ = 25.0°, θ_min_ = 1.6°
ω scans	*h* = −9→10
Absorption correction: multi-scan (*ABSCOR*; Higashi, 1995)	*k* = −9→11
*T*_min_ = 0.215, *T*_max_ = 0.340	*l* = −16→15
6095 measured reflections	

### Refinement

**Table:**

Refinement on *F*^2^	Primary atom site location: structure-invariant direct methods
Least-squares matrix: full	Secondary atom site location: difference Fourier map
*R*[*F*^2^ > 2σ(*F*^2^)] = 0.025	Hydrogen site location: inferred from neighbouring sites
*wR*(*F*^2^) = 0.065	H-atom parameters constrained
*S* = 1.08	*w* = 1/[σ^2^(*F*_o_^2^) + (0.0293*P*)^2^ + 1.6634*P*] where *P* = (*F*_o_^2^ + 2*F*_c_^2^)/3
4012 reflections	(Δ/σ)_max_ = 0.004
319 parameters	Δρ_max_ = 0.83 e Å^−^^3^
0 restraints	Δρ_min_ = −0.46 e Å^−^^3^

### Special details

**Table:**

Geometry. All e.s.d.'s (except the e.s.d. in the dihedral angle between two l.s. planes) are estimated using the full covariance matrix. The cell e.s.d.'s are taken into account individually in the estimation of e.s.d.'s in distances, angles and torsion angles; correlations between e.s.d.'s in cell parameters are only used when they are defined by crystal symmetry. An approximate (isotropic) treatment of cell e.s.d.'s is used for estimating e.s.d.'s involving l.s. planes.
Refinement. Refinement of *F*^2^ against ALL reflections. The weighted *R*-factor *wR* and goodness of fit *S* are based on *F*^2^, conventional *R*-factors *R* are based on *F*, with *F* set to zero for negative *F*^2^. The threshold expression of *F*^2^ > σ(*F*^2^) is used only for calculating *R*-factors(gt) *etc*. and is not relevant to the choice of reflections for refinement. *R*-factors based on *F*^2^ are statistically about twice as large as those based on *F*, and *R*- factors based on ALL data will be even larger.

### Fractional atomic coordinates and isotropic or equivalent isotropic displacement parameters (Å^2^)

**Table:**

	*x*	*y*	*z*	*U*_iso_*/*U*_eq_	Occ. (<1)
Pb	0.51296 (2)	0.384529 (19)	0.321343 (15)	0.03622 (8)	
S1	0.86425 (18)	0.07481 (19)	0.43924 (14)	0.0634 (4)	
S2	0.20076 (16)	−0.09836 (15)	0.28488 (12)	0.0527 (4)	
S3	0.6251 (3)	0.1416 (2)	−0.04539 (14)	0.0887 (6)	
S4	0.1267 (3)	0.5374 (2)	0.10337 (14)	0.0845 (6)	
N1	0.6815 (5)	0.2053 (5)	0.3667 (3)	0.0462 (11)	
N2	0.9129 (5)	0.3416 (6)	0.4149 (5)	0.0660 (15)	
H2A	0.9059	0.4168	0.3862	0.079*	
H2B	1.0104	0.3441	0.4233	0.079*	
N3	0.3856 (4)	0.1266 (4)	0.2963 (3)	0.0395 (9)	
N4	0.1501 (5)	0.1384 (5)	0.2314 (4)	0.0614 (14)	
H4A	0.1852	0.2369	0.2254	0.074*	
H4B	0.0971	0.0753	0.1816	0.074*	
N5	0.5432 (6)	0.2735 (5)	0.1318 (3)	0.0537 (12)	
N6	0.7467 (8)	0.1533 (8)	0.1351 (5)	0.101 (2)	
H6A	0.7241	0.1444	0.2003	0.121*	
H6B	0.8060	0.0773	0.1133	0.121*	
N7	0.3075 (5)	0.4293 (5)	0.1959 (3)	0.0450 (10)	
N8	0.1492 (5)	0.5684 (5)	0.3050 (4)	0.0566 (12)	
H8A	0.0613	0.5979	0.3009	0.068*	
H8B	0.1761	0.5443	0.3564	0.068*	
N9	0.2813 (5)	0.3333 (5)	0.4915 (3)	0.0417 (10)	
N10	0.7714 (5)	0.5968 (5)	0.2504 (4)	0.0499 (11)	
O1	0.7860 (4)	0.4915 (5)	0.2859 (4)	0.0662 (12)	
O2	0.6464 (4)	0.6191 (5)	0.2306 (3)	0.0637 (11)	
O3	0.8811 (5)	0.6758 (5)	0.2381 (4)	0.0757 (13)	
O4	0.2622 (5)	0.4100 (5)	0.4368 (4)	0.0767 (14)	
O5	0.4074 (4)	0.3241 (4)	0.5214 (3)	0.0583 (10)	
O6	0.1759 (5)	0.2579 (6)	0.5126 (4)	0.0799 (14)	
C1	0.8164 (6)	0.2223 (6)	0.4046 (4)	0.0465 (13)	
C2	0.6914 (7)	−0.0124 (7)	0.4019 (5)	0.0649 (17)	
H2	0.6586	−0.1055	0.4053	0.078*	
C3	0.6101 (6)	0.0701 (5)	0.3673 (4)	0.0417 (11)	
C4	0.4582 (6)	0.0295 (5)	0.3302 (4)	0.0406 (11)	
C5	0.3759 (6)	−0.0963 (6)	0.3282 (5)	0.0536 (14)	
H5	0.4097	−0.1719	0.3476	0.064*	
C6	0.2493 (6)	0.0720 (5)	0.2691 (4)	0.0433 (12)	
C7	0.6410 (8)	0.1928 (7)	0.0868 (5)	0.0664 (17)	
C8	0.4800 (9)	0.2367 (9)	−0.0394 (5)	0.082 (2)	
H8	0.4276	0.2442	−0.0961	0.099*	
C9	0.4521 (7)	0.2994 (6)	0.0586 (4)	0.0565 (15)	
C10	0.3355 (7)	0.3902 (6)	0.0933 (4)	0.0528 (14)	
C11	0.2503 (10)	0.4381 (8)	0.0331 (5)	0.084 (2)	
H11	0.2573	0.4198	−0.0375	0.100*	
C12	0.2007 (6)	0.5091 (6)	0.2119 (4)	0.0479 (13)	
O1W	0.8919 (7)	0.9099 (6)	0.1556 (7)	0.143 (3)	
H1A	0.8768	0.8420	0.1848	0.172*	
H1B	0.9025	0.8687	0.0946	0.172*	
O2WA	0.952 (12)	0.968 (9)	−0.012 (7)	0.46 (3)	0.50
H2C	0.8712	0.9381	−0.0459	0.553*	0.50
H2D	1.0193	0.9627	−0.0553	0.553*	0.50
O2WB	1.007 (6)	0.784 (5)	−0.045 (3)	0.46 (3)	0.50
H2E	0.9815	0.6950	−0.0507	0.553*	0.50
H2F	1.0980	0.7961	−0.0538	0.553*	0.50

### Atomic displacement parameters (Å^2^)

**Table:**

	*U*^11^	*U*^22^	*U*^33^	*U*^12^	*U*^13^	*U*^23^
Pb	0.03230 (12)	0.03302 (12)	0.04414 (12)	0.00269 (8)	0.00326 (8)	0.01255 (8)
S1	0.0507 (9)	0.0742 (11)	0.0805 (11)	0.0126 (8)	−0.0077 (8)	0.0450 (9)
S2	0.0488 (8)	0.0411 (7)	0.0670 (9)	−0.0075 (6)	0.0027 (7)	0.0187 (7)
S3	0.1077 (16)	0.1029 (15)	0.0530 (10)	0.0293 (13)	0.0292 (10)	0.0096 (10)
S4	0.1085 (16)	0.0875 (13)	0.0631 (11)	0.0429 (12)	−0.0244 (10)	0.0174 (9)
N1	0.038 (2)	0.045 (2)	0.060 (3)	0.010 (2)	0.002 (2)	0.021 (2)
N2	0.035 (3)	0.062 (3)	0.109 (5)	−0.001 (2)	−0.009 (3)	0.041 (3)
N3	0.038 (2)	0.036 (2)	0.045 (2)	0.0012 (18)	0.0018 (18)	0.0118 (18)
N4	0.047 (3)	0.051 (3)	0.091 (4)	−0.007 (2)	−0.019 (3)	0.033 (3)
N5	0.063 (3)	0.055 (3)	0.047 (3)	0.016 (2)	0.013 (2)	0.018 (2)
N6	0.114 (6)	0.131 (6)	0.068 (4)	0.079 (5)	0.023 (4)	0.017 (4)
N7	0.044 (3)	0.049 (3)	0.047 (3)	0.004 (2)	−0.002 (2)	0.022 (2)
N8	0.047 (3)	0.071 (3)	0.061 (3)	0.018 (2)	0.005 (2)	0.028 (3)
N9	0.035 (2)	0.052 (3)	0.038 (2)	0.001 (2)	0.0006 (18)	0.014 (2)
N10	0.047 (3)	0.053 (3)	0.053 (3)	0.005 (2)	0.005 (2)	0.020 (2)
O1	0.047 (2)	0.061 (3)	0.104 (4)	0.005 (2)	0.003 (2)	0.047 (3)
O2	0.050 (3)	0.075 (3)	0.076 (3)	0.013 (2)	−0.006 (2)	0.034 (2)
O3	0.054 (3)	0.075 (3)	0.113 (4)	−0.003 (2)	0.013 (3)	0.055 (3)
O4	0.069 (3)	0.078 (3)	0.112 (4)	0.033 (3)	0.034 (3)	0.064 (3)
O5	0.047 (2)	0.066 (3)	0.057 (2)	0.0002 (19)	−0.0071 (19)	0.012 (2)
O6	0.050 (3)	0.100 (4)	0.098 (4)	−0.015 (3)	−0.006 (2)	0.052 (3)
C1	0.039 (3)	0.055 (3)	0.053 (3)	0.011 (3)	0.004 (2)	0.024 (3)
C2	0.057 (4)	0.057 (4)	0.093 (5)	0.003 (3)	−0.007 (3)	0.042 (3)
C3	0.043 (3)	0.042 (3)	0.044 (3)	0.006 (2)	0.005 (2)	0.017 (2)
C4	0.041 (3)	0.037 (3)	0.045 (3)	0.006 (2)	0.008 (2)	0.013 (2)
C5	0.051 (3)	0.041 (3)	0.074 (4)	0.003 (3)	0.001 (3)	0.026 (3)
C6	0.046 (3)	0.038 (3)	0.042 (3)	−0.002 (2)	0.002 (2)	0.008 (2)
C7	0.075 (5)	0.069 (4)	0.055 (4)	0.022 (4)	0.014 (3)	0.012 (3)
C8	0.092 (6)	0.101 (6)	0.050 (4)	0.014 (5)	0.003 (4)	0.013 (4)
C9	0.068 (4)	0.055 (3)	0.046 (3)	−0.001 (3)	0.006 (3)	0.016 (3)
C10	0.068 (4)	0.045 (3)	0.045 (3)	0.006 (3)	−0.001 (3)	0.011 (2)
C11	0.123 (7)	0.090 (5)	0.044 (3)	0.032 (5)	−0.010 (4)	0.021 (3)
C12	0.048 (3)	0.042 (3)	0.055 (3)	−0.002 (3)	−0.010 (3)	0.020 (3)
O1W	0.117 (5)	0.087 (4)	0.240 (9)	−0.022 (4)	−0.075 (6)	0.087 (5)
O2WA	0.66 (8)	0.50 (7)	0.31 (4)	0.30 (6)	0.16 (4)	0.17 (4)
O2WB	0.66 (8)	0.50 (7)	0.31 (4)	0.30 (6)	0.16 (4)	0.17 (4)

### Geometric parameters (Å, °)

**Table:**

Pb—N1	2.656 (4)	N7—C12	1.311 (7)
Pb—N3	2.563 (4)	N7—C10	1.375 (7)
Pb—N5	2.535 (5)	N8—C12	1.354 (7)
Pb—N7	2.692 (4)	N8—H8A	0.8999
Pb—O1	2.704 (4)	N8—H8B	0.8393
Pb—O4	2.803 (5)	N9—O4	1.210 (6)
S1—C2	1.717 (7)	N9—O6	1.227 (6)
S1—C1	1.726 (5)	N9—O5	1.252 (5)
S2—C5	1.714 (6)	N10—O3	1.233 (6)
S2—C6	1.728 (5)	N10—O2	1.242 (6)
S3—C8	1.709 (8)	N10—O1	1.262 (6)
S3—C7	1.726 (7)	C2—C3	1.330 (7)
S4—C11	1.710 (8)	C2—H2	0.9300
S4—C12	1.720 (5)	C3—C4	1.458 (7)
N1—C1	1.319 (7)	C4—C5	1.349 (7)
N1—C3	1.395 (6)	C5—H5	0.9300
N2—C1	1.345 (7)	C8—C9	1.344 (9)
N2—H2A	0.9230	C8—H8	0.9300
N2—H2B	0.9039	C9—C10	1.474 (8)
N3—C6	1.313 (6)	C10—C11	1.338 (9)
N3—C4	1.390 (6)	C11—H11	0.9300
N4—C6	1.355 (7)	O1W—H1A	0.8534
N4—H4A	0.9949	O1W—H1B	0.8279
N4—H4B	0.8762	O2WA—O2WA^i^	1.0 (2)
N5—C7	1.313 (8)	O2WA—H2C	0.8498
N5—C9	1.391 (8)	O2WA—H2D	0.8500
N6—C7	1.323 (9)	O2WB—H2E	0.8502
N6—H6A	0.9337	O2WB—H2F	0.8500
N6—H6B	0.9558		
			
N5—Pb—N3	78.40 (14)	O3—N10—O1	119.2 (5)
N5—Pb—N1	90.14 (15)	O2—N10—O1	118.7 (5)
N3—Pb—N1	65.73 (13)	N10—O1—Pb	105.8 (3)
N5—Pb—N7	64.98 (15)	N1—C1—N2	124.6 (5)
N3—Pb—N7	89.39 (13)	N1—C1—S1	114.4 (4)
N1—Pb—N7	148.47 (14)	N2—C1—S1	121.0 (4)
N5—Pb—O1	75.24 (16)	C3—C2—S1	111.4 (5)
N3—Pb—O1	132.73 (13)	C3—C2—H2	124.3
N1—Pb—O1	75.77 (13)	S1—C2—H2	124.3
N7—Pb—O1	113.10 (13)	C2—C3—N1	115.0 (5)
C2—S1—C1	89.0 (3)	C2—C3—C4	125.6 (5)
C5—S2—C6	89.2 (3)	N1—C3—C4	119.4 (4)
C8—S3—C7	89.4 (3)	C5—C4—N3	114.7 (5)
C11—S4—C12	88.7 (3)	C5—C4—C3	125.5 (5)
C1—N1—C3	110.2 (4)	N3—C4—C3	119.7 (4)
C1—N1—Pb	133.5 (4)	C4—C5—S2	111.0 (4)
C3—N1—Pb	115.4 (3)	C4—C5—H5	124.5
C1—N2—H2A	127.5	S2—C5—H5	124.5
C1—N2—H2B	123.9	N3—C6—N4	125.3 (5)
H2A—N2—H2B	102.7	N3—C6—S2	114.5 (4)
C6—N3—C4	110.5 (4)	N4—C6—S2	120.2 (4)
C6—N3—Pb	130.0 (3)	N5—C7—N6	124.9 (6)
C4—N3—Pb	118.7 (3)	N5—C7—S3	114.5 (5)
C6—N4—H4A	115.8	N6—C7—S3	120.6 (5)
C6—N4—H4B	109.5	C9—C8—S3	110.7 (6)
H4A—N4—H4B	120.6	C9—C8—H8	124.7
C7—N5—C9	110.0 (5)	S3—C8—H8	124.7
C7—N5—Pb	129.2 (4)	C8—C9—N5	115.4 (6)
C9—N5—Pb	120.7 (4)	C8—C9—C10	126.0 (6)
C7—N6—H6A	114.4	N5—C9—C10	118.7 (5)
C7—N6—H6B	131.4	C11—C10—N7	115.5 (6)
H6A—N6—H6B	100.3	C11—C10—C9	125.7 (6)
C12—N7—C10	109.9 (5)	N7—C10—C9	118.8 (5)
C12—N7—Pb	132.8 (4)	C10—C11—S4	110.9 (5)
C10—N7—Pb	115.3 (3)	C10—C11—H11	124.5
C12—N8—H8A	112.9	S4—C11—H11	124.5
C12—N8—H8B	120.1	N7—C12—N8	124.8 (5)
H8A—N8—H8B	121.3	N7—C12—S4	115.0 (4)
O4—N9—O6	119.0 (5)	N8—C12—S4	120.3 (4)
O4—N9—O5	120.7 (5)	H1A—O1W—H1B	105.1
O6—N9—O5	120.1 (5)	H2C—O2WA—H2D	107.7
O3—N10—O2	122.1 (5)	H2E—O2WB—H2F	107.7

Symmetry codes: (i) −*x*+2, −*y*+2, −*z*.

### Hydrogen-bond geometry (Å, °)

**Table:**

*D*—H···*A*	*D*—H	H···*A*	*D*···*A*	*D*—H···*A*
N2—H2A···O1	0.92	2.08	2.884 (8)	145
N2—H2B···O4^ii^	0.90	2.33	3.209 (7)	165
N2—H2B···O6^ii^	0.90	2.31	3.057 (7)	140
N4—H4A···N7	0.99	2.19	3.168 (7)	166
N4—H4B···O1W^iii^	0.88	2.29	3.015 (8)	141
N4—H4B···O2WA^iv^	0.88	2.26	2.98 (9)	140
N6—H6A···N1	0.93	2.22	3.119 (8)	160
N6—H6B···O2WA^v^	0.96	2.29	3.12 (10)	145
N6—H6B···O1W^v^	0.96	2.10	2.929 (10)	144
N8—H8A···O3^vi^	0.90	2.17	3.027 (7)	159
N8—H8B···O4	0.84	2.13	2.916 (7)	156
O1W—H1A···O3	0.85	1.94	2.782 (8)	168
O1W—H1B···O2WA	0.83	1.97	2.54 (9)	125
O1W—H1B···O2WB	0.83	2.14	2.93 (4)	160
O2WA—H2C···N4^iv^	0.85	2.42	2.98 (9)	124
O2WA—H2D···O1W^i^	0.85	2.17	2.85 (9)	136
O2WB—H2E···S4^iv^	0.85	2.27	3.09 (5)	164
O2WB—H2F···S3^vii^	0.85	2.80	3.53 (5)	144
O2WB—H2F···N6^vii^	0.85	1.91	2.67 (5)	148

Symmetry codes: (ii) *x*+1, *y*, *z*; (iii) *x*−1, *y*−1, *z*; (iv) −*x*+1, −*y*+1, −*z*; (v) *x*, *y*−1, *z*; (vi) *x*−1, *y*, *z*; (i) −*x*+2, −*y*+2, −*z*; (vii) −*x*+2, −*y*+1, −*z*.
